# The Essential Role of Physiologic Assessment in Anomalous Aortic Origin of Coronary Arteries: Moving Beyond Purely Anatomical Evaluation

**DOI:** 10.1016/j.jscai.2025.103758

**Published:** 2025-07-23

**Authors:** David G. Rizik, Morton J. Kern

**Affiliations:** a Department of Cardiovascular Medicine, HonorHealth, Scottsdale, Arizona; b Department of Cardiology VA Long Beach, Long Beach, California

**Keywords:** anomalous coronary arteries, aortic surgery, atherosclerosis, cardiovascular Health

In this issue of the *JSCAI*, Tasouli-Drakou et al^1^ describe the case of an anomalous right coronary artery (RCA) arising from the left coronary sinus in a 59-year-old Hispanic man who presented with symptoms of chest pain. Coronary computed tomography angiography demonstrated a noncalcified plaque in the left anterior descending artery (LAD) and an anomalous RCA with a so-called “malignant intramural and interarterial course.” Also described is the coexistence of a rare bovine arch variant (in the absence of Tetralogy of Fallot), which—while anatomically novel—likely adds little to the management dilemma.

If the anomalous RCA was the presumed culprit for the patient’s anginal symptoms, it would seem that the diagnostic waters were made murky by the angiographic presence of a 90% to 95% stenosis of the proximal LAD. In the end, the patient was referred for surgical therapy: a left internal mammary artery graft to the LAD anastomosis and “unroofing” of the intramural portion of the RCA from the aortic wall.

Although the anatomical assessment using coronary computed tomography angiography was thorough, and referral to invasive catheter-based assessment of coronary anatomy appropriate, this case raises an important question: Are we doing enough when we rely solely on an anatomical evaluation to guide surgical decisions in anomalous aortic origin of coronary arteries (AAOCA)?

For AAOCAs, surgeons and cardiologists often base their decision for surgical intervention primarily on concerning anatomical features—an interarterial course, acute angle takeoff, and slit-like ostium.^2^, ^3^, ^4^, ^5^, ^6^ Although these features have traditionally guided management, emerging evidence suggests that a physiologic assessment may provide crucial additional information for clinical decision-making.

Consider the potential value of invasive physiologic testing in this case. Fractional flow reserve (FFR) measurement using adenosine and dobutamine as stressors, increasing both contractility and myocardial flow, would likely provide objective evidence of functional significance.^7^ The dynamic nature of AAOCA ischemic mechanisms of the intra-aortic wall course, deformation of the ostial orifice, and potential compression between great vessels make dobutamine FFR particularly valuable, as it can unmask functional compromise not evident at rest. An FFR <0.80 with either or both stressors (adenosine/dobutamine) strengthens the case for surgery, whereas normal values might have supported a more conservative management with close monitoring.

Intravascular ultrasound (IVUS) could have added another crucial dimension to the evaluation. IVUS enables direct visualization of the vessel wall and dynamic compression, potentially identifying cases where angiographic features often overestimate or underestimate functional significance. The ability to perform IVUS during pharmacologic stress would provide unique insights into the dynamic nature of coronary compression that static imaging alone cannot capture.

The authors’ approach reflects current standard practice, and they should be commended for their careful anatomical assessment; however, their case highlights a significant opportunity to review the current approaches to AAOCA and the integration of physiologic testing to improve outcomes. Consider the important change in management had this patient’s AAOCA been negative for ischemia and the anginal syndrome relieved with a simple LAD stent.

The benefits of a comprehensive evaluation would include the objective quantification of functional compromise using both adenosine and dobutamine and confirmation of dynamic compression using IVUS during dobutamine stress by directly visualizing vessel wall mechanics and geometry, and ultimately, better identifying which patients will most likely benefit from surgery.

Given the potential benefits, why is physiologic testing not standard practice in AAOCA evaluation? Several factors likely contribute to the underutilization, including limited experience with these techniques, lack of standardized protocols, and absence of confidence in established threshold values for intervention in this unique anatomy. These challenges should motivate us to address the knowledge gaps for diagnosis rather than accept a purely anatomical assessment alone. As our understanding of AAOCA grows, the integration of functional testing and imaging will refine our approach to these challenging cases. Several unanswered questions limiting the adoption of IVUS and physiology for AAOCA management are evident. For example:

• What are the appropriate FFR thresholds for AAOCA?
• Can physiological testing help identify which patients truly need surgical intervention?
• What is the optimal protocol for stress testing during invasive assessment?
• How do findings on IVUS correlate with clinical outcomes?

Over the past decade, invasive physiologic assessment (FFR/nonhyperemic pressure ratios) and anatomic interrogation with optical coherence tomography and IVUS now represent state-of-the-art management of coronary artery disease; these should be considered to complement the diagnosis of AAOCA, particularly in cases where the decision for surgical intervention is not clear based on anatomy alone. In the absence of the physiologic significance of the RCA, this case would certainly been simplified (or at least, less complex) as a single-vessel, proximal LAD, catheter-based intervention. Multicenter studies incorporating standardized physiologic testing protocols will be particularly valuable in advancing our understanding of AAOCA.

The interventional treatment of coronary artery disease has matured and suggests that the application of imaging and physiology to the field of AAOCA management among other complex anatomic presentations (eg, CTO, fistula, and myocardial bridge) stands at a crossroads. Although anatomical assessment remains fundamental, the addition of physiology testing may provide the objective data needed to optimize patient selection for surgery. Future cases like this would benefit from such comprehensive evaluation, potentially leading to more personalized and evidence-based management decisions.

## Peer review statement

Associate Editor David G. Rizik and Section Editor Morton J. Kern had no involvement in the peer review of this article and have no access to information regarding its peer review. Full responsibility for the editorial process for this article was delegated to Editor-in-Chief Alexandra J. Lansky.
